# Supplementary material for: Anomalous polarization enhancement in a van der Waals ferroelectric material under pressure
Source: Nat Commun. 2023 Jul 18;14:4301. doi: 10.1038/s41467-023-40075-6 (PMC10354068; doi:10.1038/s41467-023-40075-6)
Supplement: Supplementary file 1 — Supplementary Information [file 41467_2023_40075_MOESM1_ESM.pdf]

Supplementary Information

Anomalous polarization enhancement in a van der Waals ferroelectric material under pressure

Xiaodong Yao,[1]† Yinxin Bai,[1]† Cheng Jin,[2]† Xinyu Zhang,[1] Qunfei Zheng,[1] Zedong Xu,[1] Lang Chen,[1] Shanmin Wang,[1] Ying Liu,[1] Junling Wang,[1,3]* Jinlong Zhu[1]*

[1] *Department of Physics, Southern University of Science and Technology, Shenzhen 518055, China.*
[2] *Center for High Pressure Science and Technology Advanced Research (HPSTAR), Beijing, 100094, China.*
[3] *Guangdong Provincial Key Laboratory of Functional Oxide Materials and Devices, Southern University of Science and Technology, Shenzhen, China.*

†: These authors contributed equally to this work.
*: corresponding authors: jwang@sustech.edu.cn (Junling Wang);
zhujl@sustech.edu.cn (Jinlong Zhu)

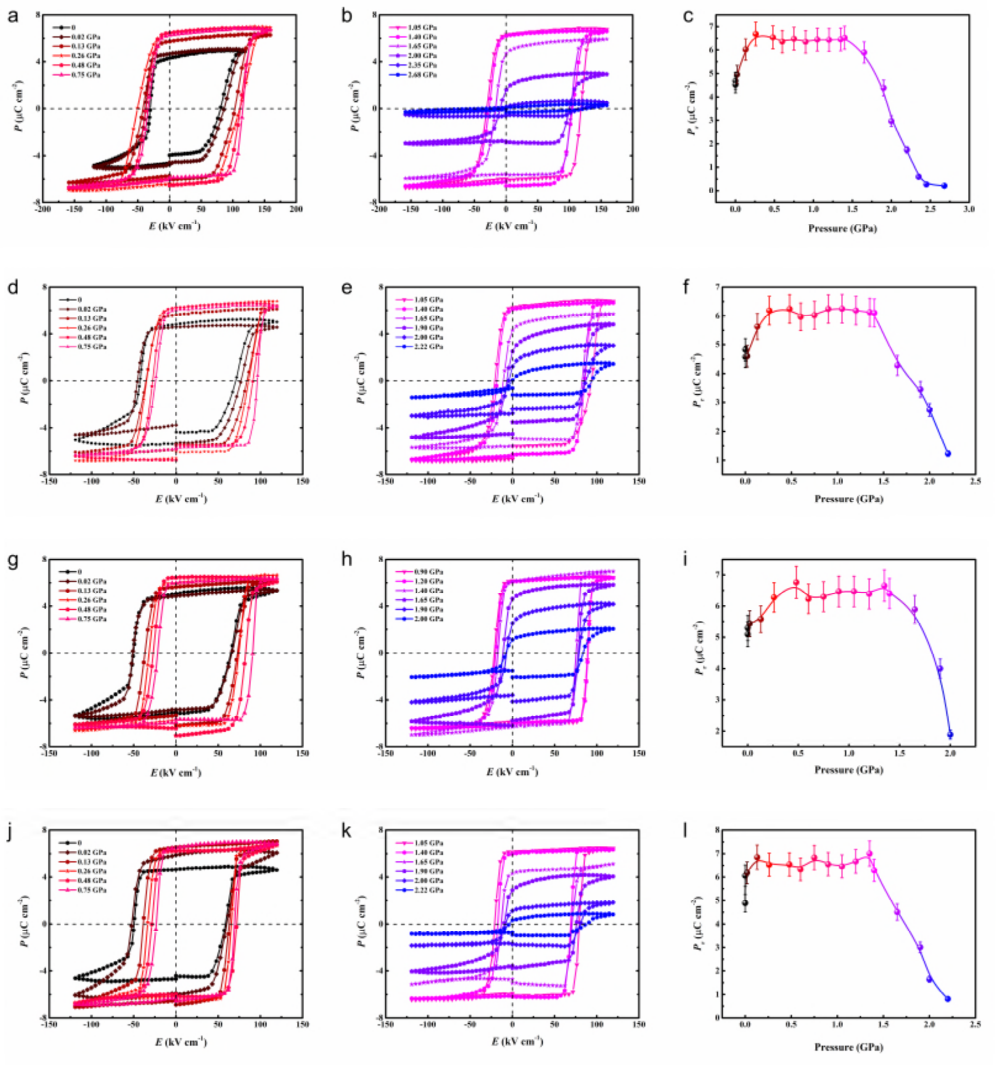

Figure S1 Polarization-electric (P-E) field hysteresis loops measured at 500, 200, 100 and 50 Hz. (a) and **(b) P-E** loops measured at representative pressures and 500 Hz, **(c)** The evolution of remanent polarization with pressure; **(d)** and **(e) P-E** loops measured at representative pressures and 200 Hz, **(f)** The evolution of remanent polarization with pressure; **(g)** and **(h) P-E** loops measured at representative pressures and 100 Hz, **(i)** The evolution of remanent polarization with pressure; **(j)** and **(k) P-E** loops measured at representative pressures and 500 Hz, **(l)** The evolution of remanent polarization with pressure. The error bars were estimated from the electrode areas.

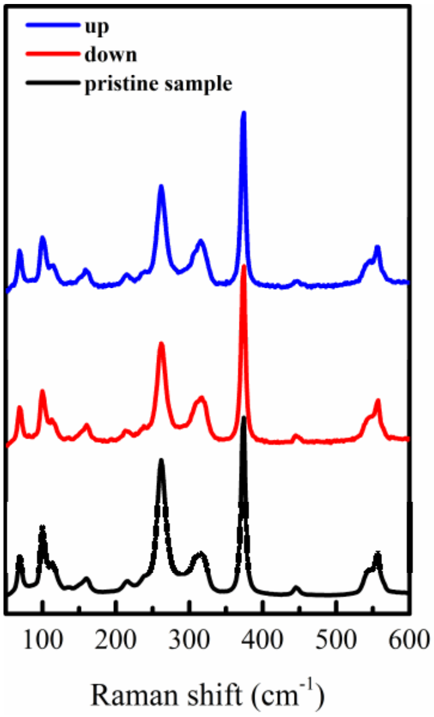

Figure S2 Raman spectra of pristine (black line), downward-polarized (red line) and upward-polarized (blue line) samples, respectively.

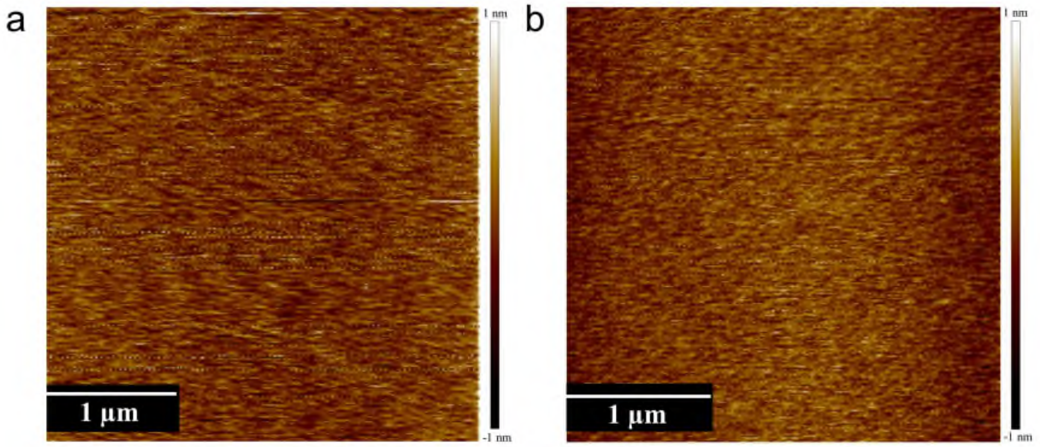

Figure S3 Surface analysis before and after the pressure study (a) AFM topography of a pristine CIPS single crystal. The Ra that is arithmetic average of absolute values is 0.137 nm. (b) AFM topography of the decompressed sample, the Ra is 0.113 nm.

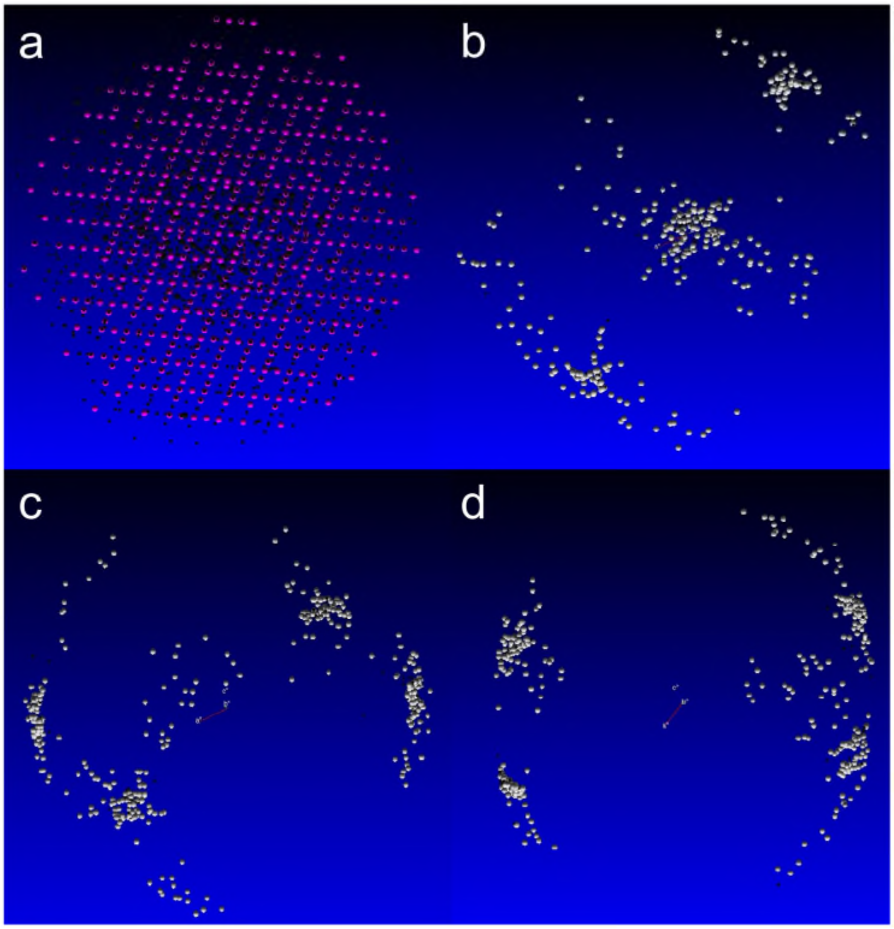

Figure S4 Single-crystal X-ray diffraction study of CIPS. The diffraction spots obtained under **(a)** ambient conditions, **(b)** 0.80 GPa, **(c)** 1.40 GPa, and **(d)** 1.80 GPa. APEX3 software was used to determine the cell parameters and volumes.

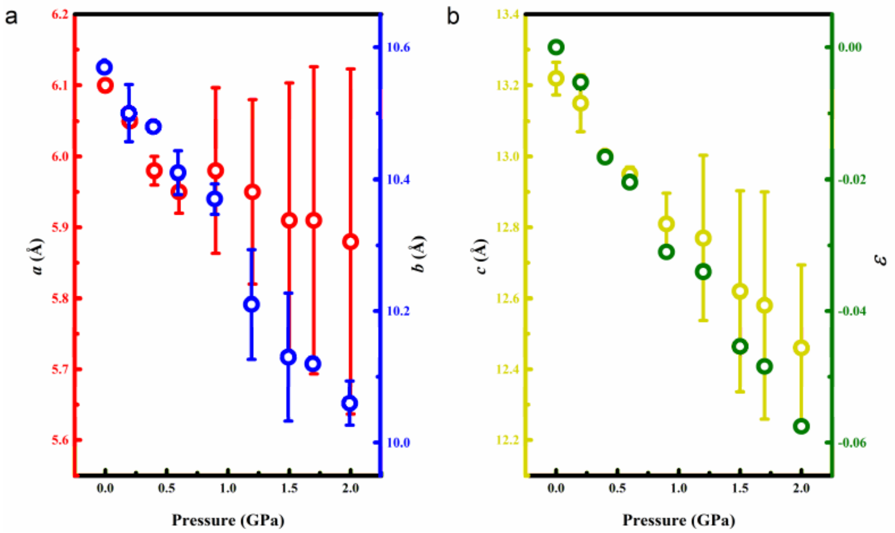

Figure S5 Pressure-dependent cell parameters. The *a*, *b* and *c*-axis of CIPS with pressure in **(a)** and **(b)**, the *ε* in (b) describes the change in the *c*-axis strain with pressure, the error bars were estimated from the APEX3 software.

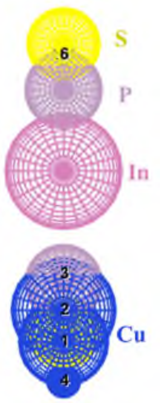

Figure S6 The reduced atom model for CIPS from Ref. [1]. The electric dipoles were calculated by using the relative negative and positive charge positions, and divided S charges proportionally to Cu, In and P forming dipoles respectively.

Table S1 Raman peaks and vibration modes in CIPS. [2]

Raman shift	Displacement patterns
72 cm^{-1}	out-of-plane Cu (polar for A' and antipolar for A'' displacements in the adjacent layers) + out-of-plane S vibration
104 cm^{-1}	rigid out-of-plane displacement of P-P dimers (in-phase phase in adjacent layers) + In displacements opposite to that of P-P dimers
116 cm^{-1}	in-plane displacement of Cu + In + S, out-of-plane S vibration
162 cm^{-1}	out-of-plane P-P + in-plane S vibration
216 cm^{-1}	in-plane Cu + P, and out-of-plane S vibration
238 cm^{-1}	in-plane Cu + P, and out-of-plane S vibration
264 cm^{-1}	in-plane S vibration
315 cm^{-1}	in-plane P + S vibration
375 cm^{-1}	out-of-plane P + in-plane S vibration
448 cm^{-1}	out-of-plane P + out-of-plane S vibration
549 cm^{-1}	in-plane P-P stretching + in-plane S vibration
558 cm^{-1}	in-plane P-P + in-plane S vibration

Table S2 A list of the projection of atomic position vector in a unit cell along the Z direction and occupancies. [1]

Atom	Cu (4)$^{1+}$	Cu (1)$^{1+}$	Cu (2)$^{1+}$	Cu (3)$^{1+}$	Cu (6)$^{1+}$	In^{3+}	P^{4+} (1)
z_i (Å)	4.27	4.72	5.09	5.50	8.03	6.69	5.40
Occupancy	12%	32%	37.3%	7.9%	8%	100%	100%
Atom	P^{4+} (2)	S (1)	S (2)	S (3)	S (4)	S (5)	S (6)
z_i (Å)	7.62	4.79	4.80	4.83	8.13	8.15	8.19
Occupancy	100%	100%	100%	100%	100%	100%	100%

Supplementary References

1. L. You, Y. Zhang, S. Zhou, A. Chaturvedi, S. A. Morris, F. Liu, L. Chang, D. Ichinose, H. Funakubo, W. Hu, T. Wu, Z. Liu, S. Dong, J. Wang, Origin of giant negative piezoelectricity in a layered van der Waals ferroelectric. *Science advances* **5**, eaav3780 (2019).

2. S. N. Neal, S. Singh, X. Fang, C. Won, F. T. Huang, S.W. Cheong, K. M. Rabe, D. Vanderbilt, J. L. Musfeldt, Vibrational properties of $CuInP_2S_6$ across the ferroelectric transition. *Physical Review B* **105**, 075151 (2022).
